# Mucocèle de la muqueuse buccale

**DOI:** 10.11604/pamj.2020.35.140.21079

**Published:** 2020-04-29

**Authors:** Soukaina Essaket, Fouzia Hakkou, Saliha Chbicheb

**Affiliations:** 1 Département d’Odontologie Chirurgicale, Faculté de Médecine Dentaire de Rabat, Université Mohammed V, Rabat, Maroc

**Keywords:** Mucocèle, glandes salivaires accessoires, traitement d’une mucocèle, Mucocele, minor salivary glands, mucocele treatment

## Abstract

Les mucocèles buccales sont des pathologies tumorales bénignes des glandes salivaires accessoires de la muqueuse buccale. La localisation la plus fréquente de ces lésions est la muqueuse labiale. Etiologiquement, elles sont de deux types: le premier est dû à une rupture de l’épithélium de la glande déversant de la salive dans l’espace extra-glandulaire, et forment un pseudo kyste (mucocèle par extravasation); le second est causé par blocage de l’évacuation salivaire par prolifération épithéliale du conduit excréteur, réalisant un vrai kyste salivaire (kyste de rétention). Il existe diverses modalités thérapeutiques, l’exérèse chirurgicale conventionnelle reste la stratégie la plus efficace où la récidive est la moins fréquente. A travers une observation clinique, une mise au point sur cette lésion est faite, illustrée par un cas clinique pris en charge dans le Service d’Odontologie Chirurgicale, Centre de Consultation et de Traitement Dentaire (CCTD), Rabat, Maroc.

## Abstract (en)

Mucoceles of the oral mucous membrane are benign tumoral diseases of the minor salivary glands of the oral mucous membrane. They most commonly occur in the labial mucosa. They can be caused by a break in the epithelium of the gland secreting saliva in the extra-glandular space and forming a pseudo cyst (extravasation mucoceles) or by a block in salivary flow due to an epithelial proliferation of the excretory duct forming salivary cyst (retention cyst). Various therapeutic approaches can be used. Traditional surgical resection is the most effective strategy with low recurrence rate. We here report a clinical case in order to give an update on this lesion. The patient involved in the study was treated in the Department of Dentistry and Oral Surgery, CCTD, Rabat, Morocco.

## Introduction

Les mucocèles ou les kystes mucoïdes sont définis comme des cavités remplies de mucus pouvant apparaître dans la cavité buccale. Ce sont des lésions relativement fréquentes de la muqueuse buccale résultant d'une altération des glandes salivaires accessoires due à une accumulation de mucus. Le développement d’une cavité remplie de mucus se fait selon deux mécanismes distincts: l’extravasation et la rétention. L’extravasation correspond à la fuite de mucus issu des canaux excréteurs ou acini des glandes salivaires dans le tissu adjacent, l’extravasation est le mécanisme principal de production de mucocèles de la muqueuse buccale avec traumatisme comme facteur initiateur. La rétention (beaucoup moins fréquente) est la conséquence d’une sténose dans les canaux excréteurs, responsable d’une expulsion inefficace de la salive entrainant la dilatation des canaux et gonflement en surface [1].

## Patient et observation

**Figure 1:**
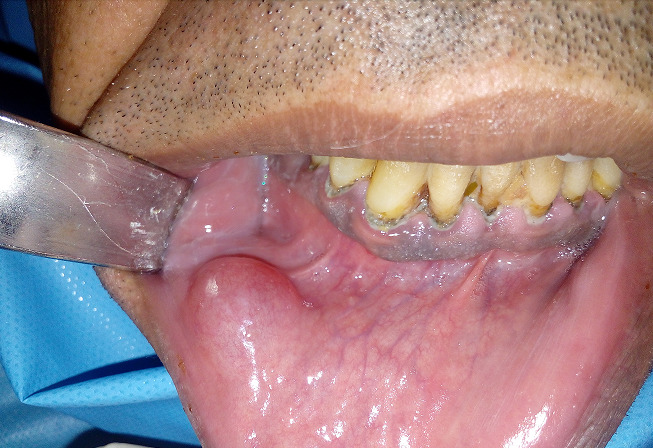
Aspect de la lésion

**Figure 2:**
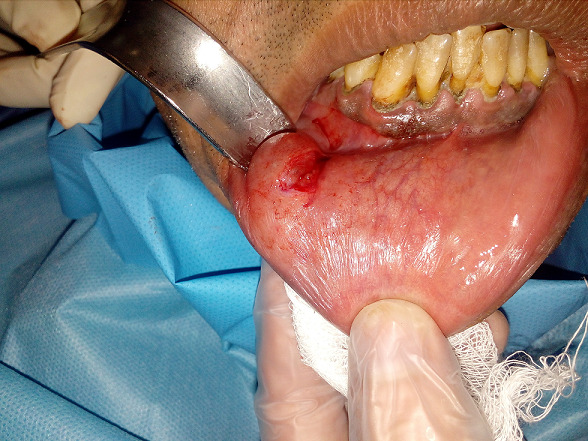
Incision initiale montrant l’aspect translucide de la lésion

**Figure 3:**
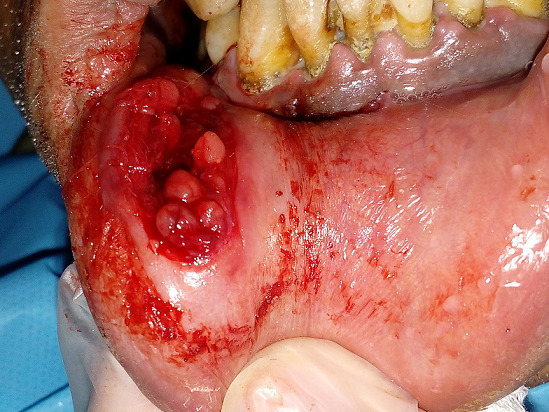
Excision de la mucocèle et les glandes salivaires accessoires dans le champ opératoire

**Figure 4:**
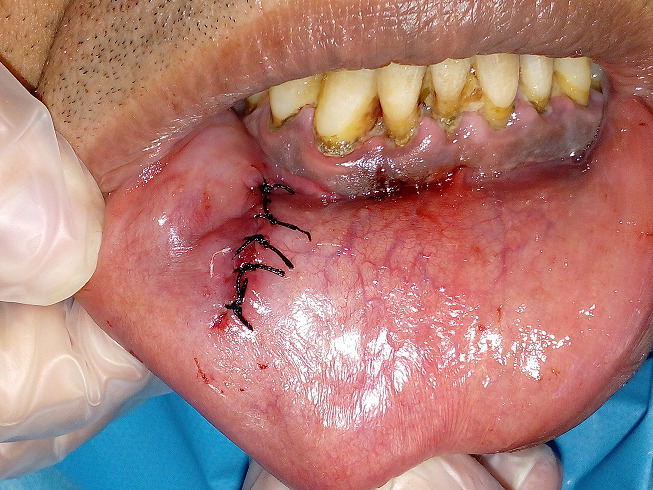
Vue postopératoire avec sutures

Il s’agit du patient M, âgé de 43 ans, en bon état de santé générale, qui a consulté pour une tuméfaction de la lèvre inférieure du côté droit. L’interrogatoire a révélé une tuméfaction de la lèvre inférieure d’évolution progressive depuis un an. A l’inspection, nous avons constaté la présence d’une tuméfaction de forme ovoïde au niveau de l’hémi lèvre inférieure droite en regard de 33-34, mesurant 2cm de longueur et 1,5 cm de large. Son aspect est bleuâtre. A la palpation, la tuméfaction est de consistance molle et mobile par rapport au plan profond. La palpation des aires ganglionnaires est sans anomalie notable (Figure 1). Compte-tenu de la localisation, de la croissance progressive et de l’aspect clinique, le diagnostic de mucocèle labiale inférieure a été évoqué, le traitement a consisté en une exérèse chirurgicale sous anesthésie locale. Après anesthésie locale et exposition du champ opératoire, l’incision initiale a été réalisée sur la ligne faîtière et la muqueuse superficielle a été disséquée prudemment (Figure 2). L’exérèse du nodule a ensuite été réalisée au bistouri lame 15 sous contrôle visuel permanent, afin d´éviter la section accidentelle d’un rameau nerveux (Figure 3). L’aspect clinique de la pièce opératoire semblait conforter l’hypothèse diagnostique de mucocèle. La fermeture du site opératoire a été réalisée à l’aide de sutures discontinues sans tension (Figure 4). L’analyse anatomopathologique retrouvait une cavité remplie de salive mucoïde en bordure de laquelle est clairement visible la glande salivaire accessoire causale, confirmant le diagnostic de mucocèle labiale. La cicatrisation à 2 semaines puis à 8 semaines n’a amené aucune bride cicatricielle résiduelle.

## Discussion

Les mucocèles sont des lésions bénignes relativement fréquentes de la muqueuse buccale, affectant essentiellement les glandes salivaires accessoires. La lèvre inférieure est le site de prédilection (60 à 80 %), car étant la zone la plus à risque de trauma notamment au niveau des dents cuspidées. Tandis que les autres sites typiques sont la joue, la face ventrale de la langue, le plancher de la bouche et la région rétromolaire [1]. Ces kystes sont le plus souvent de faux kystes traumatiques par extravasation salivaire, plutôt que des kystes par rétention. Les mucocèles par extravasation représentent 80 à 90% des mucocèles, contre 10 à 20% de vrais kystes par rétention. Le kyste mucoïde par extravasation affecte préférentiellement le sujet masculin entre la deuxième et la troisième décennie, par contre celui par rétention touche surtout le sujet âgé [2]. Elle porte le nom du mucocèle de Blandin-Nuhn lorsqu’elle est située à la face ventrale de la langue [3]. Et de grenouillette quand la localisation est au niveau du plancher buccal (Le terme de grenouillette vient de la ressemblance entre ce kyste et le ventre d’une grenouille). Elle est le plus souvent unilatérale, son origine vient de la glande sublinguale [4]. Les facteurs étiologiques comprennent les traumatismes accidentels (comme celles dus au brossage dentaire et aux habitudes de morsure ou de succion), ainsi que l’obstruction partielle ou totale des canaux par une micro-lithiase salivaire [1].

Les mucocèles sont des lésions asymptomatiques, mais dans quelques cas, lorsqu'elles se présentent sous forme de lésions multiples et récurrentes, elles peuvent provoquer une douleur intense [5]. Cliniquement, il s’agit d’une tuméfaction kystique fluctuante ou rénitente, de taille variable de quelque millimètre à quelque centimètre de diamètre. Au début, la lésion est de consistance molle avec un aspect bleuté. La lésion prend un aspect translucide au cours de son évolution. La consistance molle disparaît quand la lésion est profonde ou ancienne pour devenir dure. La palpation est indolore [2]. Le diagnostic se discute avec d’autres tumeurs, notamment des glandes salivaires accessoires, du kyste dermoïde, d’un angiome veineux ainsi que d’autres lésions de la muqueuse buccale [2]. En cas de mucocèle de rétention, la cavité kystique présente une paroi épithéliale sous forme d’une rangée de cellules cuboïdes ou plates produites du canal excréteur des glandes salivaires [6]. En l’absence de traitement, des épisodes de diminutions et d’augmentations de taille de la lésion peuvent apparaître à cause d’une alternance de rupture de la paroi et de reprise du kyste [3]. Le traitement classique repose sur l’ablation totale du kyste. L’exérèse doit se faire avec une grande douceur pour assurer l’élimination de la totalité de la lésion sans perforation de la poche kystique. Une autre approche chirurgicale avec le laser CO_2_ permet une cicatrisation sans rétraction. L’incision, la cautérisation acide et les exérèses incomplètes sont les principales causes de récidives [2].

La lésion d’un rameau labial du nerf mentonnier est une considération importante lors de toute chirurgie du versant muqueux de la lèvre inférieure. En effet, dans de nombreux cas, ces rameaux peuvent s’avérer être très superficiels. Une étude anatomique d’Alsaad *et al*. en 2003 sur le nerf mentonnier a montré qu’il n’existe aucune voie d’abord de la lèvre inférieure exempte de risque de lésion de ce nerf, compte tenu de sa topographie superficielle. Ce risque augmente avec la taille de la mucocèle, d’où l’importance de son diagnostic précoce afin de pouvoir en faire l’exérèse dans les meilleures conditions possibles [7]. Autres techniques opératoires ont été décrites, telles que l'injection intra-lésionnelle de corticostéroïdes, la cryochirurgie, la micro-marsupialisation et la marsupialisation afin de prévenir les dommages des structures anatomiques voisines [1]. La micro marsupialisation est considérée comme un traitement alternatif idéal chez les enfants, car elle est rapide, simple et donne de bons résultats, avec un risque de récidive de 14% [1]. Le but de cette technique est de drainer le mucus ce qui permet de réduire la taille de la lésion. Elle consiste à faire passer un fil de soie épais à travers la lésion à son plus grand diamètre, puis réaliser un nœud chirurgical. La suture est enlevée après 7 à 10 jours, le temps nécessaire pour la disparition de la mucocèle [6]. Certaines études comme celle de García *et al*. en 2009 ont suggéré l’utilisation de la cryochirurgie ou l’injection intralésionnelle de corticostéroïdes dans le traitement de ces lésions. Cependant, en raison du taux élevé de récidive la procédure chirurgicale conventionnelle reste préférable [4]. L'étude anatomopathologique est cruciale pour confirmer le diagnostic. Les mucocèles d'extravasation sont des pseudokystes sans parois définies. Le mucus extravasé est entouré d'une couche de cellules inflammatoires, puis d'un tissu de granulation réactif constitué de fibroblastes, provoqué par une réaction immunitaire. Même s'il n'y a pas de revêtement épithélial autour de la muqueuse, le tissu de granulation est bien encapsulé [1].

## Conclusion

Le kyste mucoïde concerne essentiellement les glandes salivaires accessoires. Son siège préférentiel est la muqueuse labiale inférieure. La chirurgie d’exérèse au large de la tumeur reste le traitement de choix malgré l’existence d’autres techniques plus modernes et plus tolérées par les patients.

## Conflits d’intérêts

Les auteurs ne déclarent aucun conflit d'intérêts.
